# A new frameshift mutation in L1CAM producing X‐linked hydrocephalus

**DOI:** 10.1002/mgg3.1031

**Published:** 2019-11-22

**Authors:** Weiqi Kong, Xueyan Wang, Jing Zhao, Min Kang, Na Xi, Shengmei Li

**Affiliations:** ^1^ Department of Prenatal Diagnosis Sichuan Provincial Hospital for Women and Children Chengdu China; ^2^ Department of image Sichuan Provincial Hospital for Women and Children Chengdu China; ^3^ Department of gynecology Sichuan Provincial Hospital for Women and Children Chengdu China

**Keywords:** frameshift mutation, L1CAM, X‐linked hydrocephalus

## Abstract

**Background:**

X‐linked hydrocephalus (XLH), characterized by mental retardation and bilateral adducted thumbs, often come out to be a genetic disorder of *L1CAM*. It codes the protein L1 cell adhesion molecule (L1CAM), playing a crucial role in the development of the nervous system. The objective of the study was to report a new disease‐causing mutation site of *L1CAM*, and gain further insight into the pathophysiology of hydrocephalus.

**Methods:**

We collect the samples of a couple and their second hydrocephalic fetus. Then, the whole‐exome sequencing and in‐depth mutation analysis were performed.

**Results:**

The variant c.2491delG (p.V831fs), located in the exon 19 of L1CAM (chrX:153131214), could damage the L1CAM function by producing a frameshift in the translation of fibronectin type‐III of L1CAM.

**Conclusion:**

We identified a novel disease‐causing mutation in *L1CAM* for the first time, which further confirmed *L1CAM* as a gene underlying XLH cases.

## Graphical abstract

We identified a novel disease‐causing mutation in L1CAM by first time, which further confirmed L1CAM as a gene underlying XLH cases.

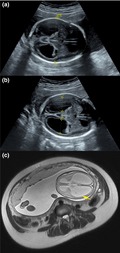

## 1 INTRODUCTION

Hydrocephalus, the abnormal accumulation of intracranial cerebrospinal fluid (CSF), is a common malformation of fetuses. Accompanied by other structural brain lesions, it affects approximately one in every 1,000 children born (Tully & Dobyns, 2014; Warf, 2005). The pathogenesis of this process remains to be fully elucidated; nonetheless, a few points are established.

A large part of hydrocephalic patients show the existence of chromosome abnormalities. Researchers has proved that there were mutations in *L1CAM* (OMIM 308840)(Marin et al., 2015; Patzke, Acuna, Giam, Wernig, & Sudhof, 2016), which code the protein L1 cell adhesion molecule (L1CAM), a neuronal cell adhesion molecule belonging to the immunoglobulin superfamily (IgSF) and playing a key role in the development of the nervous system (Chang, Rathjen, & Raper, 1987; Rathjen & Schachner, 1984).

Mutations in *L1CAM* can result in different X‐linked neurological syndromes, known as L1 syndrome (Lyonnet et al., 1992; Schrander‐Stumpel, Legius, Fryns, & Cassiman, 1990).

The diagnosis of hydrocephalus is mainly based on the result of ultrasound detection, which is neither precise nor timely. Thus, the use of genetic sequencing is increasingly popular and important in recent years. The purpose of the study was to report a new disease‐causing mutation site of *L1CAM*, making a small step forward in the pathogenesis of hydrocephalus.

## 2 MATERIALS AND METHODS

### 2.1 Ethical compliance

The research was approved by the Institutional Committee for the Protection of Human Subjects (Institutional Review Board of Sichuan Provincial Hospital for Women and Children), and all patients signed the informed consent.

### 2.2 Sample collection

The blood samples of the parents and the tissue of their fetus were collected and kept at −80°C.

### 2.3 Mutation analysis

Genomic DNA was extracted from tissue and blood samples according to standard protocols. Applied Biosystems 3730xl DNA Analyzer was used to sequence the result of PCR amplification. Then, we found out the sites that need to be sequenced on the peak of Sanger sequencing, specific primers were designed according to the site information on USCS via Prime Primer 5, and to confirm whether they have variation. The library was further constructed by using Roche SeqCap EZ MedExome Enrichment kit and sequenced on an Illumina HiSeq X machine. Raw reads were mapped to the human reference genome GRCh37/hg19 using BWA (v0.7.12‐r1039) (Li & Durbin, 2009), and the SAM files were transformed to BAM files and sorted by using SAMtools (v0.1.18). Then Picard v1.134 (http://broadinstitute.github.io/picard/) was used to mark duplicate reads. Variants were called by GenomeAnalysisTK (GATK v3.7) (McKenna et al., 2010) and annotated by ANNOVAR (2016Jul16 version). The Exome Aggregation Consortium (ExAC Version 0.3.1), Genomes 1,000 Project, ESP6500, and other public database were used to filter the variants. The candidate pathogenic mutations were verified by Sanger sequencing.

## 3 RESULTS

**Figure 1:**
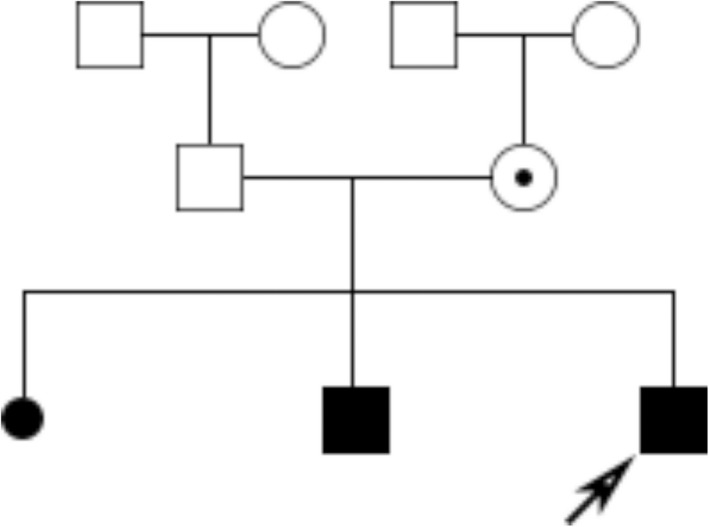
Pedigree of the family

A 25‐year‐old woman was referred to our department for having one spontaneous abortion and two voluntary terminations of pregnancy due to fetal hydrocephalus. Blood samples of this couple and tissue of the last hydrocephalic fetus were collected. The familial pedigree was consistent with X‐linked recessive inheritance (Figure 1).

During the first pregnancy, a natural abortion happened around 11 weeks of gestation. As for the second pregnancy, a fetal ultrasound scan at 24+ weeks of gestation proved the presence of hydrocephalus, and the woman required an interruption of pregnancy.

**Figure 2:**
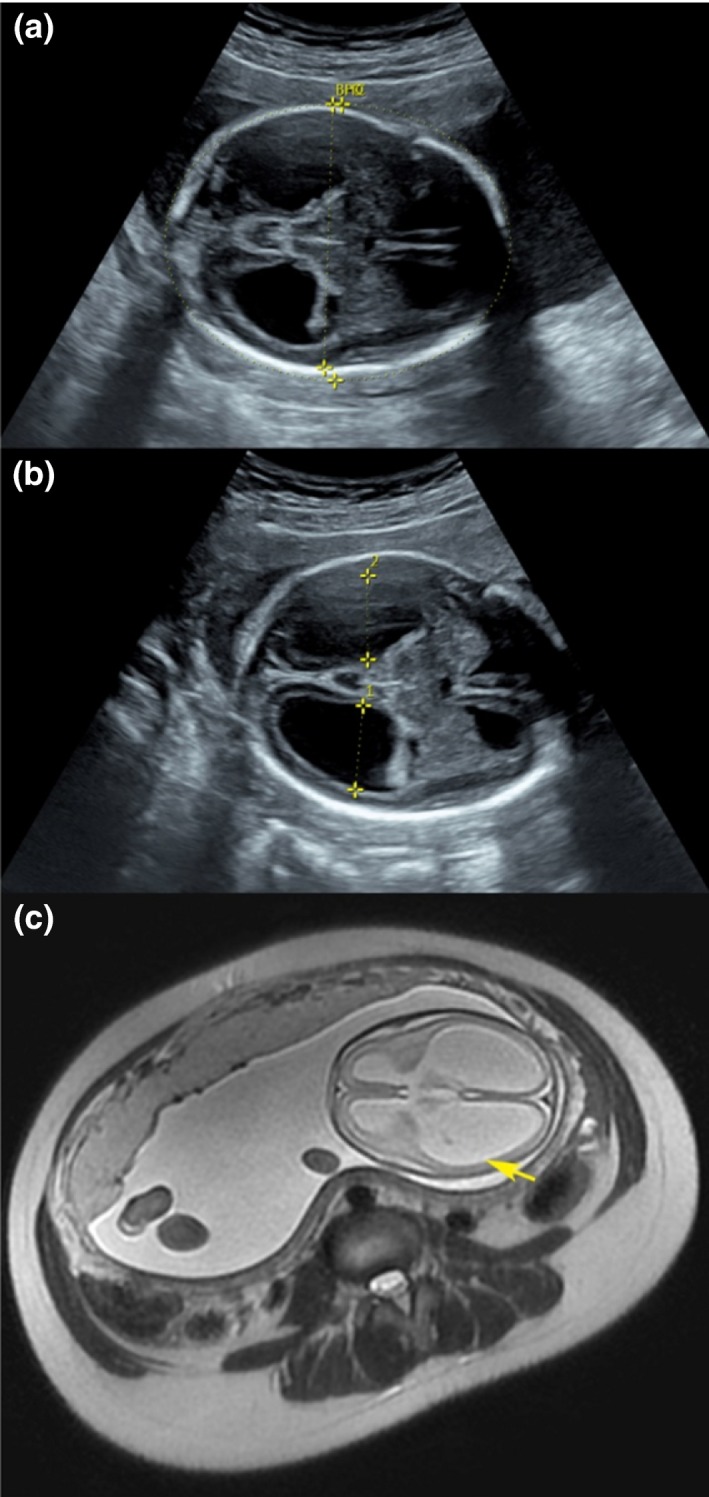
The ultrasound scan and magnetic resonance imaging (MRI) scan. (a) The ultrasound scan, BPD = 6.54 cm, HC = 24.36 cm; (b) The ultrasound scan, 1D = 2.09 cm, 2D = 2.08 cm; (c) MRI scan

The third pregnancy was similar to the second one. The ultrasound scan evaluation at 25 + 4 gestation weeks revealed the bilateral ventriculomegaly with dilatation of the third ventricle and polyhydramnios. The magnetic resonance imaging (MRI) further proved the presence of callosal agenesis and lissencephaly (Figure 2).

**Figure 3:**
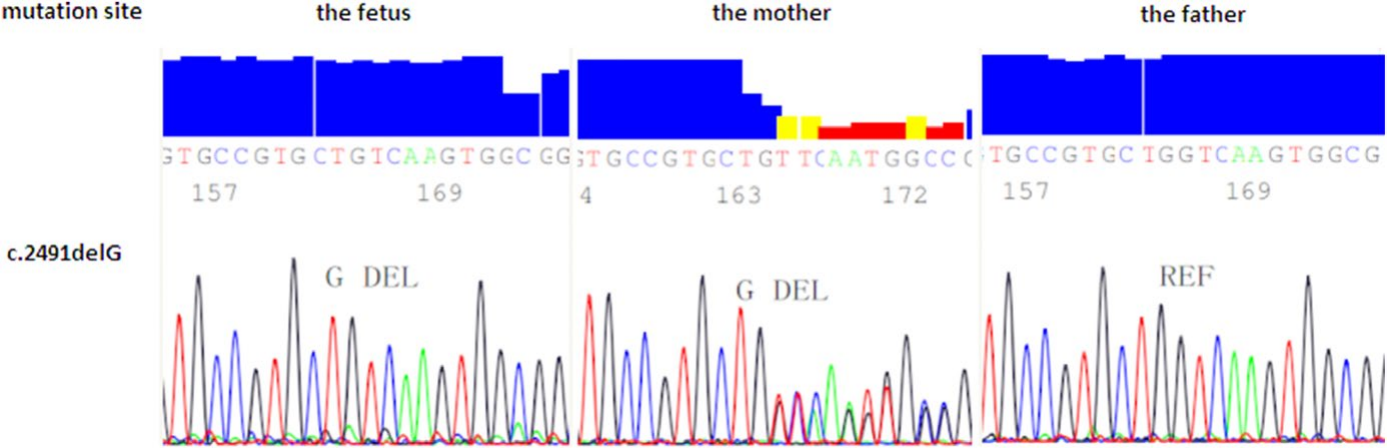
Sequence analysis results (GenBank ID number M77640.1)

After collecting the samples of the couple and the third fetus, we used the whole‐exome sequencing and in‐depth mutation analysis to find the potential causes. The variant was confirmed in DNA extracted from the fetus and mother (Figure 3). Potential variants were called by Genome Analysis TK, public databases were used to filter the variants, and the effects of these variants were annotated by ANNOVAR programs.

The proband was found to be hemizygous for *L1CAM* with a mutation NM_000425.5:c.2491del:p.(Val831Serfs*20) and the mother was found to be heterozygote. Computational analysis predicted that this was a frameshift mutation, located in the exon 19 of *L1CAM* (chrX:153131214), coding the fibronectin type‐III of L1CAM. The mutation will lead to the translation errors of amino acid and an early translation termination, belonging to the loss‐of‐function mutation. Moreover, the ClinGen haploinsufficiency score and pLI in ExAC of *L1CAM* was 3 and 1, respectively, (https://www.ncbi.nlm.nih.gov/projects/dbvar/clingen/clingen_gene.cgi?sym=L1CAM&subject=, and http://exac.broadinstitute.org/gene/ENSG00000198910), suggesting a strong relationship between the loss‐of‐function mutation and disease. In addition, by now, this mutation had not been reported in gnomAD or 1,000 Genomes Project yet. Besides, according to OMIM, diseases related to *L1CAM* are X‐linked recessive, and the phenotype was consistent with that of the proband. The genetic pattern was consistently with “the L1 syndrome.” According to the guide of ACMG (American society of medical genetics and genomics), with the evidence of a PVS1, a PM2, and a PP4, it was an X‐linked and pathogenic mutation (Figure 3).

## 4 DISCUSSION

Hydrocephalus, including X‐linked hydrocephalus (XLH), often comes out to be a genetic disorder, characterized by mental retardation and bilateral adducted thumbs (Okamoto et al., 2004). The features of XLH include the enlargement of both third and lateral ventricles, agenesia of corpus callosum, atrophy of corticospinal descending pathways in the pons and medulla, and spasticity of upper and mostly lower limbs (Itoh & Fushiki, 2015; Tonosaki et al., 2014). Despite of its unclear pathogenesis, most cases reported the strong link between mutations of *L1CAM* and XLH.

L1CAM consists of six immunoglobulin, five fibronectin III‐like domains, a single pass transmembrane domain, and a short cytoplasmic domain (Moos et al., 1988). It produces a variety of molecular and cellular effects, crucial to brain development (Chang et al., 1987; Rathjen & Schachner, 1984). The dysfunction of L1CAM can lead to “the L1 syndrome,” which is X‐linked, including hydrocephalus with stenosis of the Sylvius aqueduct (HSAS; phenotype MIM number 307000), MASA (mental retardation, aphasia, spastic paraplegia, adducted thumbs) syndrome (phenotype MIM number 303350), complicated hereditary spastic paraplegia type 1 (SPG1, phenotype MIM number 303350), and agenesis of the corpus callosum (phenotype MIM number 308840) (Basel‐Vanagaite et al., 2006).

According to Vos et al.’s report (Vos et al., 2010), 85% of hydrocephalus fetus were facing *L1CAM* mutation when they had three or more L1 syndrome‐related morphological alterations, and more than one affected relative. Ferese et al. (2016) reported that a splicing mutation (NM_000425.4:c.1267 + 5delG) in *L1CAM*, which produced the skipping of exon 10, could result in hydrocephalus. In Liebau's study (Liebau, Gal, Superti‐Furga, Omran, & Pohl, 2007), a mutation at the beginning of intron 18 of *L1CAM* was related to the agenesis of corpus callosum, adducted thumbs, hydrocephalus, and mental retardation. Hübner et al. (2004) found out that a mutation of *L1CAM* in two unrelated families resulted in a frame shift due to insertion of the first 10 bp of intron 5 in the mature mRNA of L1CAM, leading to a largely truncated protein. In our study, we found a NM_000425.5:c.2491del:p.(Val831Serfs*20) variant, located in the exon 19 of *L1CAM* (chrX:153131214), that could damage the L1CAM function by producing a frameshift in the translation of fibronectin type‐III of L1CAM, resulting in the bilateral ventriculomegaly with dilatation of the third ventricle, polyhydramnios, callosal agenesis, and lissencephaly.

In summary, we identified a novel XLH‐causing mutation NM_000425.5:c.2491del:p.(Val831Serfs*20) in L1CAM for the first time. The L1CAM mutations are manifold, and most of them are unique for each family (Vos et al., 2010). The more disease‐causing mutations we found, the more accurate predictions we are able to make.

## CONFLICT OF INTEREST

The authors declared that they have no conflicts of interest to this work. We declare that we do not have any commercial or associative interest that represents a conflict of interest in connection with the work submitted.

## AUTHORS' CONTRIBUTIONS

Dr. Xueyan Wang and Dr. Weiqi Kong had full access to all of the data in the study and take responsibility for the integrity of the data and the accuracy of the data analysis.
